# Inter-prescriber variability in the decision to prescribe antibiotics to febrile patients attending primary care in Myanmar

**DOI:** 10.1093/jacamr/dlaa118

**Published:** 2021-01-19

**Authors:** Myo Maung Maung Swe, Elizabeth A Ashley, Thomas Althaus, Yoel Lubell, Frank Smithuis, Alistair R D Mclean

**Affiliations:** 1 Myanmar Oxford Clinical Research Unit, Yangon, Myanmar (MOCRU), Yangon, Myanmar; 2 The Centre for Tropical Medicine and Global Health, Nuffield Department of Medicine, University of Oxford, Oxford, UK; 3 Lao-Oxford-Mahosot Hospital-Wellcome Trust Research Unit (LOMWRU), Vientiane, Laos; 4 Mahidol Oxford Tropical Medicine Research Unit (MORU), Faculty of Tropical Medicine, Mahidol University, Bangkok, Thailand; 5 Medical Action Myanmar, Yangon, Myanmar

## Abstract

**Background:**

Most antibiotic prescribing occurs in primary care. Even within the same health facility, there may be differences between prescribers in their tendency to prescribe antibiotics, which may be masked by summary data. We aimed to quantify prescriber variability in antibiotic prescription to patients with acute fever in primary care clinics in Myanmar.

**Methods:**

We conducted a secondary analysis of prescribing data from 1090 patient consultations with 40 prescribing doctors from a trial investigating the effect of point-of-care C-reactive protein (CRP) tests on antibiotic prescription for acute fever. We used multilevel logistic regression models to assess inter-prescriber variability in the decision to prescribe antibiotics.

**Results:**

The median odds ratio (MOR) in the unadjusted model was 1.82 (95% CI: 1.47–2.56) indicating that when two prescribers from this population are randomly selected then in half of these pairs the odds of prescription will be greater than 1.82-fold higher in one prescriber than the other. The estimated variability from this sample of prescribers corresponds to a population of prescribers where the top 25% of prescribers will prescribe antibiotics to over 41% of patients while the bottom 25% will prescribe antibiotics to less than 23% of patients. Inter-prescriber variation in antibiotic prescribing remained after adjustment for patient characteristics and CRP information (*P *<* *0.001).

**Conclusions:**

Despite sharing the same management guidelines, there was substantial inter-prescriber variation in antibiotic prescription to patients with acute fever. This variation should be considered when designing trials and stewardship programmes aiming to reduce inappropriate antibiotic prescribing.

## Introduction

Antimicrobial resistance (AMR) is a growing problem and becoming a threat to public health globally.^1^ Bacteria are becoming increasingly resistant to antibiotics and unnecessary antibiotic use in human and agricultural sectors is fuelling the process.^2^ Global antibiotic consumption increased by 35% between 2000 and 2010 with increased use of last resort antibiotic classes.^3^ Consumption of antibiotics is associated with development of antibiotic resistance.^4^^,^^5^ The majority of antibiotic prescriptions for humans occurs in primary care facilities, particularly for patients with febrile illness^6–9^ and respiratory tract infections.^10^^,^^11^ There are concerns that most of these prescriptions may be unnecessary.^12^ Identifying febrile patients who could benefit from receiving antibiotics is challenging for healthcare workers, especially in resource poor settings where diagnostic facilities are limited.^13^ A situational analysis in Myanmar in 2014 found that, on average, 47% of outpatients received an antibiotic across primary care facilities (ranging from 34% to 54%) and 87% of those with upper respiratory tract infections received an antibiotic across primary care facilities (ranging from 73% to 96%).^14^ Antimicrobial stewardship activities are in the early stages of development and implementation in Myanmar.^15^

Even within the same health facility, there may be substantial differences between prescribers in their tendency to prescribe antibiotics.^16^^,^^17^ Consequently, summary data of antibiotic prescriptions at a health facility may mask the variation that exists among the prescribers in that facility. Where considerable inter-prescriber variation exists, a health facility may have doctors who prescribe at the appropriate level, alongside doctors who are overprescribing and doctors who are under-prescribing. Antimicrobial stewardship programmes in health facilities that target behaviour of high prescribing doctors such as peer comparison and feedback have the potential to reduce antibiotic overuse where there is high variation in antibiotic prescription among doctors.^18^^,^^19^ Studies have shown variation of antibiotic prescription among primary care physicians and GPs in different countries^20–22^ but data from Southeast Asia and low- and middle-income settings are limited. A failure to account for inter-prescriber variation in the design and analysis of antimicrobial stewardship studies may lead to inefficient study designs, inappropriate analysis and incorrect conclusions.

To assess inter-prescriber variability and patient factors that may influence the decision to prescribe we performed a secondary analysis on data collected from a randomized control trial on management of acute febrile patients in primary care facilities conducted in 2016–17 in Myanmar.^23^ The objectives of this analysis were to quantify the amount of inter-prescriber variation in antibiotic prescription for patients with acute fever in one organization with uniform management guidelines, to investigate whether patient characteristics could explain variation among prescribing doctors and to quantify associations between patient characteristics and the doctor’s decision to prescribe antibiotics.

## Methods

### Study dataset

This is a secondary analysis of data generated by a randomized controlled trial investigating the effect of point-of-care C-reactive protein (CRP) testing on antibiotic prescription for febrile patients in primary care facilities in Myanmar that showed point-of-care CRP testing reduced the proportion of antibiotic prescriptions in the intervention group compared with the control group.^23^ In brief, patients over 1 year of age presenting with an acute fever to three primary care clinics operated by Medical Action Myanmar (MAM) (a non-governmental organization) and one outpatient department of a township hospital were recruited into the study. Fever was defined by tympanic temperature >37.5 °C (or axillary temperature >37°C) or history of fever ≤14 days. In the original trial, patient recruitment was stratified by age (children defined as <12 years and adults defined as ≥12 years) and they were randomized with a ratio of 1:1:1 into one of the three groups; two CRP-guided intervention groups and one routine care control group. Study staff on site carried out study-related procedures including recruitment and point-of-care CRP testing to patients in the intervention groups prior to the patients consulting with prescribing doctors. Then for the intervention groups, the CRP result was communicated to the prescribing doctors as ‘low CRP’ or ‘high CRP’ using a CRP cut-off of 20 mg/L in intervention group A and 40 mg/L in intervention group B. If the patient was in the control group then the prescribing doctors were given no information about the CRP status of the patient. Point-of-care CRP testing was not performed for patients in the control group, where prescribing doctors were advised to manage patients as routine practice. Due to the nature of the study design, prescribing doctors were not masked to the intervention or control status of the patients. However, they were masked to the allocation of intervention group A or B, and they were not informed of the exact value of the CRP result.

We followed-up patients at day 5 and day 14 to assess recovery from the illness. While the trial primary outcome was the percentage of patients prescribed an antibiotic up to and including the day 5 visit, the current analysis was restricted to antibiotic prescription at the first consultation (day 0) in the three primary care clinics as our aim was to estimate the inter-prescriber variability of prescribers working in the same organization (with the same management guidelines) who are providing care to the same patient population. We included all three study groups from three MAM clinics in the analysis. We did not include the township hospital in the analysis because of different management guidelines and different patient population.

MAM clinics are located in industrial slums approximately 20 km outside of Yangon downtown area. The clinics open 7 days a week from 9.00 to 17.00 and provide a range of healthcare services including general medical care, HIV prevention and treatment, reproductive healthcare and malnutrition care. All services are provided free of charge. Patients recruited to this study were seen by the next available general doctor. MAM used clinical guidelines created by Médecins Sans Frontières for management of patients in the clinics.

### Statistical analysis

Patient characteristics were summarized with *n*/*N* (%) for categorical or median (25th, 75th percentile) for continuous variables. The data had a multilevel structure; each prescriber could have multiple patient consultations. To quantify the variation in antibiotic prescribing between prescribers we fit a multilevel logistic regression model (prescriber only model) with the prescriber decision to prescribe antibiotics (yes/no) as the outcome and prescriber-specific random effects (prescriber-varying intercept). We provide estimates and 95% CIs for the between-prescriber variance and the corresponding variance partition coefficient (VPC) and median odds ratio (MOR). The VPC represents the proportion of the total observed variation in antibiotic prescription that is attributable to between‐prescriber variation. The MOR can be interpreted as a 0.5 probability that when two prescribers are randomly selected from the population, the odds of prescription will be greater than the value of MOR in one prescriber than the other.^24^

We assessed prescriber-specific residuals graphically. Using the distribution of prescription probability for the population of prescribers that would result from the estimate of the intercept and variance, we calculated the implied percentiles of prescription probability amongst the population of prescribers and visualized this graphically.

To explore whether the variation in antibiotic prescribing could be explained by differences in patients’ clinical presentation and to quantify the association between patient characteristics and the decision to prescribe antibiotics, we fit a multilevel logistic regression model (patient characteristics adjusted model) with prescriber-specific random effects and eight patient characteristics (selected *a priori* as potential covariates associated with the decision to prescribe) and study site as fixed effects. The following patient characteristics were included as covariates: age (categorical: <12 and ≥12 years as in the trial analysis); gender (male or female); presence of comorbidities (yes or no); prior antibiotic use (yes or no); fever on presentation (yes or no); self-reported duration of fever at presentation (categorical: ≤2 and >2 days); CRP information (categorical: no CRP test, high CRP, low CRP); self-reported duration of respiratory symptoms at presentation (categorical: no respiratory symptoms, ≤3 days and >3 days). Variance, VPC and MOR were calculated and the adjusted association between patient characteristics and the decision to prescribe antibiotics were present as adjusted OR estimates and 95% CI. We used STATA Version 15 (StataCorp, TX, USA) for the analysis.

### Ethical approval

Protocol, informed consent forms and study related documents were reviewed and approved by Oxford Tropical Research Ethics Committee (OxTREC) and the research ethics committee of the Department of Medical Research (DMR), Myanmar. This trial was registered in ClinicalTrials.gov (registration no. NCT02758821).

## Results

### Patient characteristics

The dataset for analysis consisted of 1090 patients with acute fever; 561 children (1 to <12 years) and 529 adolescents/adults (≥12 years) enrolled into the trial between November 2016 and June 2017 in three clinics in Myanmar. There were 40 prescribers who conducted a median (minimum–maximum) of 10 (1–125) patient consultations (Figure S1, available as Supplementary data at *JAC-AMR* Online). Patient characteristics are shown in Table 1 and Figure S2. Overall, the prescribers prescribed antibiotics to 34% (372/1090) of patients. Prescribers made the decision to prescribe antibiotics more frequently to patients who had documented fever on presentation (39%) than those who did not (29%); to patients with fever duration >2 days (44%) than patients with fever duration ≤2 days (26%); and to patients who had respiratory symptoms at presentation (34% for ≤3 and 54% for >3 days duration) than those who did not (23%).

**Table 1.** Patient characteristics and proportion of patients who received an antibiotic prescription

Subgroup	Total patients, *n*/*N* (%)	Proportion of patients prescribed antibiotics
All patients	1090	0.34
Study sites		
Clinic A (HTY A clinic)	416/1090 (38%)	0.32
Clinic B (HTY B clinic)	343/1090 (31%)	0.36
Clinic C (SPT clinic)	331/1090 (30%)	0.34
Age		
≥12 years	529/1090 (49%)	0.36
<12 years	561/1090 (51%)	0.32
Gender		
male	487/1090 (45%)	0.35
female	603/1090 (55%)	0.33
Comorbid diseases^a^		
no	954/1090 (88%)	0.34
yes	145/1090 (13%)	0.31
Prior antibiotic use		
no	1033/1090 (95%)	0.33
yes	57/1090 (5.2%)	0.49
Fever on presentation^b^		
no	480/1084 (44%)	0.29
yes	604/1084 (56%)	0.39
Duration of fever^c^		
≤2 days	565/1073 (53%)	0.26
>2 days	508/1073 (47%)	0.44
Respiratory symptoms duration^d^		
no respiratory symptoms	311/1088 (29%)	0.23
≤3 days	598/1088 (55%)	0.34
>3 days	179/1088 (16%)	0.54
CRP information		
no CRP test	365/1090 (33%)	0.38
low CRP	521/1090 (48%)	0.15
high CRP	204/1090 (19%)	0.76

See Figure S2 for the distribution of patient age and gender by prescriber.

a Diabetes, hypertension, asthma, hepatitis B/C infection, HIV, congenital heart disease, gastritis, rheumatic fever, ureteric stone, epilepsy, alcoholism, G6PD deficiency, ischaemic heart disease.

b Data not recorded in six patients.

c Data not recorded in 17 patients.

d Data not recorded in two patients.

### Inter-prescriber variability

**Figure 1.**
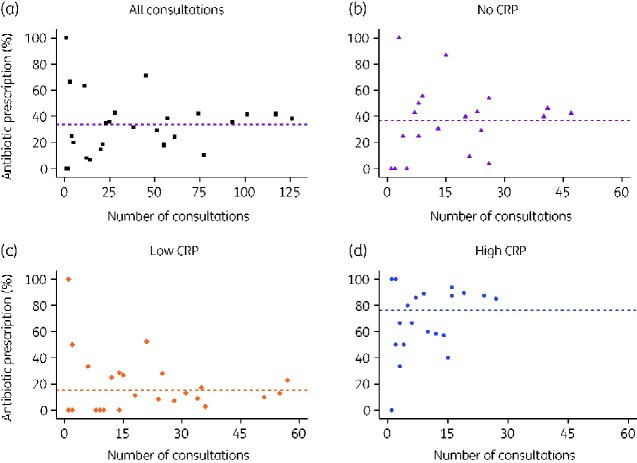
Observed percentage of patient consultations prescribed antibiotics by prescribers. (a) All consultations, (b) consultations with no CRP information, (c) consultations with low CRP and (d) consultations with high CRP. Dashed horizontal lines represents the percentage of all patients receiving an antibiotic prescription for the corresponding category. Note that some points overlap: 11 prescribers saw 1 patient and did not prescribe antibiotics and 5 prescribers saw 1 patient and did prescribe antibiotics. (For a figure with these data stratified by clinic see Figure S3).

There was substantial variation between doctors in the observed percentage of their patients who received an antibiotic prescription (Figure 1a and Figure S3). The variation persisted when consultations were stratified by CRP information (Figure 1b–d).

**Figure 2.**
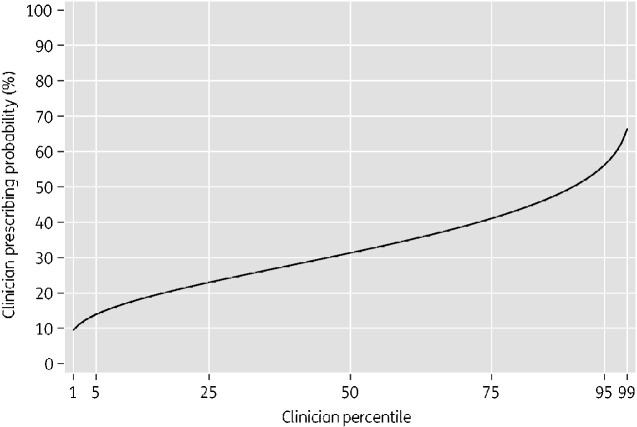
Predicted variability in prescribing among clinicians. Distribution function of predicted prescribing probability of clinicians presented as a percentage. Under this distribution, a clinician at the 75th percentile of prescribing prescribes antibiotics to 41% of their consultations while a clinician at the 25th percentile prescribes antibiotics to 23% of their consultations. The distribution is derived from the estimate of the intercept (−0.784) and estimate of the variance (0.396) from the prescriber-only model.

Table 2 shows variance components of two multilevel logistic regression models: a prescriber-only model and a patient characteristics adjusted model. In the prescriber-only model there was strong evidence of inter-prescriber variation (*P *<* *0.001, likelihood ratio test of multilevel logistic regression compared with ordinary logistic regression). The variance was 0.39 (95% CI: 0.16–0.97) and the VPC was 0.11 (95% CI: 0.05–0.23) implying that 11% of the residual variation in prescribing an antibiotic was attributable to unobserved prescriber characteristics. Caterpillar plots of prescriber residuals are shown in Figures S4 and S5. The MOR was 1.82 (95% CI: 1.47–2.56), which can be interpreted as a 50% probability that when two prescribers are randomly selected from this population the odds of prescription will be more than 1.82-fold higher in one prescriber than the other. Using the model estimate of variance and the intercept (−0.784 log odds) we graphically displayed the distribution of prescribing probability among the prescriber population (Figure 2). Under this distribution, the top 5% of doctors prescribe antibiotics in more than 56 of every 100 consultations, the top 25% in more than 41 of every 100 consultations, the bottom 25% in fewer than 23 of every 100 consultations and the bottom 5% in fewer than 13 of every 100 consultations.

**Table 2.** Variance components of model 1 (prescriber-only model) and model 2 (patient characteristics and study site adjusted model)

	Estimate (95% CI)	
	Prescriber-only model	Patient characteristics and site adjusted model
Variance	0.39 (0.16–0.97)	0.71 (0.29–1.70)
VPC	0.11 (0.05–0.23)	0.18 (0.08–0.34)
MOR	1.82 (1.47–2.56)	2.23 (1.68–3.47)

See Figure S4 for distribution of prescriber residuals in a caterpillar plot of the prescriber only model and Figure S5 for the adjusted model.

To examine if inter-prescriber variation could be explained by patient characteristics, and to assess the contribution of patient characteristics to the decision to prescribe antibiotics, we fitted an adjusted multilevel logistic regression model including eight patient characteristics and site (Table 3). After adjustment, there was still strong evidence of inter-prescriber variation (*P *<* *0.001, likelihood ratio test). Variance, VPC and MOR were not reduced after adjustment for patient characteristics (Table 2), suggesting that differences in patients’ characteristics do not explain variation in antibiotic prescription among prescribers.

**Table 3.** Adjusted OR from multilevel logistic regression model with prescriber as random effects adjusting for patient characteristics and study site

Patient characteristic	Adjusted OR (95% CI)	*P* value
Study sites		
Clinic A (HTY A clinic)	reference	
Clinic B (HTY B clinic)	1.58 (0.72–3.47)	0.25
Clinic C (SPT clinic)	1.18 (0.56–2.49)	0.67
Age		
≥12 years	reference	
12 years	0.76 (0.51–1.11)	0.16
Gender		
male	reference	
female	1.04 (0.76–1.44)	0.79
Comorbidities		
no	reference	
yes	1.20 (0.74–1.95)	0.46
Prior antibiotic use		
no	reference	
yes	2.82 (1.40–5.69)	0.004
Fever on presentation		
no	reference	
yes	1.65 (1.17–2.32)	0.004
Duration of fever		
≤2 days	reference	
2 days	2.00 (1.42–2.82)	<0.001
Respiratory symptoms duration (days)		
no symptoms	reference	
≤3 days	1.85 (1.25–2.75)	0.002
3 days	3.56 (2.13–5.94)	<0.001
CRP information		
no CRP test	reference	
low CRP	0.24 (0.16–0.34)	<0.001
high CRP	5.98 (3.84–9.32)	<0.001

### Relationship between patient characteristics and decision to prescribe antibiotics

Several patient characteristics were associated with the doctor’s decision to prescribe an antibiotic after adjustment (Table 3). Prescribers made the decision to prescribe antibiotics more frequently to patients with duration of fever >2 days than patients with duration of fever ≤2 days [OR = 2.00 (95% CI: 1.42–2.82)] and to patients with respiratory symptoms than those without [OR = 1.85 (95% CI: 1.25–2.75) for respiratory symptoms duration ≤3 days and OR = 3.56 (95% CI: 2.13–5.94) for >3 days]. Doctors prescribed antibiotics more often to patients with prior antibiotic use [OR = 2.82 (95% CI: 1.40–5.69)] and patients who had fever on presentation [OR = 1.65 (95% CI: 1.17–2.33)]. Prescribers were more likely to prescribe antibiotics to patients with high CRP than those with no CRP [OR = 5.98 (95% CI: 3.84–9.32)] and less likely to prescribe antibiotics to patients with low CRP [OR = 0.24 (95% CI: 0.16–0.34)] than patients with no CRP information. There was no evidence of any clustering of observed patient factors not used for stratification by prescribers (*P *>* *0.05 for all).

## Discussion

Using a dataset from a trial investigating the impact of point-of-care CRP testing on antibiotic prescription in patients with acute fever, we assessed variation in antibiotic prescription between primary care doctors. We used multilevel logistic regression models to quantify the amount of variation and investigated whether differences in patient characteristics could explain this variation. First, we showed that there was a considerable inter-prescriber variation in antibiotic prescription among the clinic doctors. Second, we observed that the variation remained after adjusting for patient characteristics, study site and CRP test results, suggesting that differences in patient clinical presentation did not explain the variation. This is consistent with the patient flow of the clinics investigated; all of the prescribers in this study were seeing patients from the general population. It is important to note that patient population in the dataset is relatively homogeneous as the trial recruited participants based on a set of eligibility criteria.^23^ In addition, all the prescribing doctors are working in the same clinics under the same management guidelines and seeing the same patient population. The decision of whether to prescribe antibiotics or not seems to depend in part on the preferences of the individual prescribing doctor rather than solely a reflection of patient clinical presentation and CRP information.

The variation in antibiotic prescription in our study is consistent with findings from other primary care settings. In the present study, the estimated MOR was 1.8 in the prescriber-only model, implying that when two prescribers are randomly selected from this population, in 50% of these pairings, the odds of prescription will be more than 1.8-fold higher in one prescriber than the other. This is consistent with studies of primary care prescribing tendencies in other settings: 1.7-fold for antibiotic prescription among family physicians in Canada and 3-fold for antibiotic prescription for acute rhinopharyngitis among GPs in France.^20^^,^^25^ A study carried out in the USA demonstrated a high variability of antibiotic prescribing among paediatricians for respiratory tract infections in emergency care and primary care settings.^26^ Substantial variation in high-risk non-steroidal anti-inflammatory drug prescribing among GPs in Scotland has also been noted.^27^ Similarly, a cross-sectional study carried out in 457 GPs across six European countries showed that prescribing style remained an important source of variation in antibiotic prescription for patients with sore throat after adjusting for patient and GP characteristics.^28^ Prescribing empirical antibiotics is not an easy task and there is often genuine uncertainty about the best course of action; one study noted that two infectious disease specialists disagreed with each other 29% of the time for prescription of antimicrobials for hospitalized ICU patients.^29^

In addition to a high CRP result, four patient characteristics were significantly associated with increased odds of a prescriber deciding to prescribe antibiotics: the patient having prior antibiotic use, the patient having fever on presentation, duration of patient fever and patient respiratory symptoms. A study done in over 5000 children with fever in 28 outpatient departments across 11 European countries showed association between a patient’s age, fever duration, CRP level, chest X-ray findings and focal abnormalities with increased antibiotic prescription.^6^ In our study, the association between patient age and antibiotic prescription had a wide 95% CI that included no association and so future research would be needed to further investigate any association and yield a more precise estimate. A study on childhood febrile illness in primary care in Malaysia showed an overall antibiotic prescription percentage of 37% with duration of fever >7 days and higher temperature as independent patient predictors for antibiotic prescription.^30^

We observed substantial variation in antibiotic prescription among 40 prescribers with 1090 patient consultations. One strength of our study is that all prescribers were working in the same organization with uniform management guidelines seeing a relatively homogeneous trial patient population, so the estimate of clinician variability is unlikely to be attributed to differences in clinical setting and management. We estimated the variation using multilevel models and presented the variability graphically and via MORs, which are interpretable to policymakers and clinicians. An advantage of presenting the median OR is that the amount of variability can be measured and interpreted in a manner similar to ORs.^31^

Our study has some limitations. We did not have a sufficient sample size to estimate association between prescriber characteristics (demography, practice years) and tendency to prescribe antibiotics. Therefore, we were not able to explore prescriber’s characteristics such as gender, years of practice, previous training and working practices that could explain some of the variability in prescription. Many of the doctors had only been practising in this clinic setting for less than 1 year and our findings should be interpreted in light of the clinician population, patient population and clinic setting. Some of the prescribing clinicians in our study are junior doctors and the variability in prescription among them might be different from other clinicians with several years of experience and practices in different patient population and different clinical settings. As patients in this study were recruited in the context of a clinical trial, the generalizability of these findings is limited to the patient population recruited; antibiotic prescribing to febrile patients not eligible for the trial was not captured.

Our study findings provide further evidence of variation in the decisions of primary care doctors in low- and middle-income countries to prescribe antibiotics to patients with acute febrile illness. The considerable prescriber variation in the tendency to prescribe antibiotics has important consequences for the design of trials seeking to influence prescribing behaviour. When planning and analysing a study that aims to estimate the impact of an intervention on a healthcare professional’s decision to prescribe, the clustered nature of patient–prescriber consultations must be considered. Ignoring prescriber variability and assuming all consultations are independent observations will lead to an overestimate of the power of the study and an underestimate of the anticipated uncertainty of the effect. A failure to appreciate the clustered nature of patient consultations within prescribers can lead to investigators inadvertently conducting a cluster randomized control trial without sufficient sample size and power if they assign prescribers to an intervention or control arm for the duration of the study.^32^ Failure to account for the existence of inter-prescriber variability in the analysis of these trials can lead to an inflated type 1 error rate and CIs with incorrect coverage, thereby generating false conclusions.^33–35^

Policymakers should appreciate that a single rate or proportion of antibiotic prescription from a clinic, hospital or country is a summary measure of many prescribers with different prescribing tendencies. A single clinic with an overall rate of antibiotic prescribing viewed as acceptable may in fact have some prescribers who are over-prescribing and some prescribers who are under-prescribing. An understanding of the variation in prescribing at the prescriber level may help target interventions appropriately. If it is only a subset of prescribers that are over-prescribing, then interventions targeting those prescribers may be more effective than interventions that aim to reduce prescription among all prescribers. If a population of prescribers has substantial variability in their tendency to prescribe, an intervention that aims to reduce prescription rates for all prescribers may reduce over-prescribing at the cost of increasing under-prescribing in healthcare workers who already prescribe at low rates. Antibiotic stewardship programmes targeting high prescribers should be considered as potential interventions to reduce unnecessary antibiotic use.

In conclusion, we found substantial inter-prescriber variability in antibiotic prescription for acute febrile illness and this variation could not be explained by differences in patient clinical presentations. Studies of interventions that aim to reduce antibiotic prescribing that fail to acknowledge prescriber variation run the risk of lacking sufficient power, applying inappropriate analyses and ultimately drawing false conclusions. Policymakers need to consider that a group of clinicians may be made up of both over-prescribers and under-prescribers, and antimicrobial stewardship interventions should take this into account.

## Supplementary Material

dlaa118_Supplementary_Data
