# Supplementary material for: Inter-prescriber variability in the decision to prescribe antibiotics to febrile patients attending primary care in Myanmar
Source: JAC Antimicrob Resist. 2021 Jan 19;3(1):dlaa118. doi: 10.1093/jacamr/dlaa118 (PMC7814214; doi:10.1093/jacamr/dlaa118)
Supplement: dlaa118_Supplementary_Data [file dlaa118_supplementary_data.docx]

# Supplementary data

***Figure S1: Histogram showing distribution of number of consultations per prescriber***


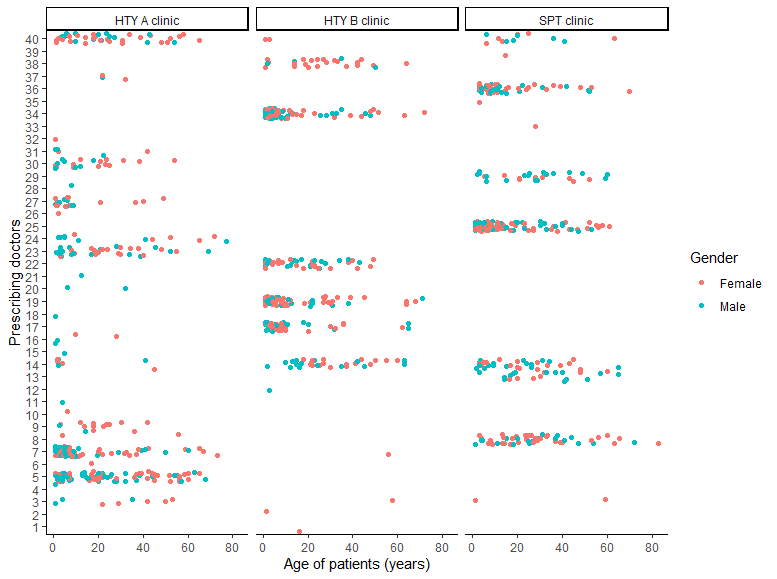


***Figure S2: Dot plot showing distribution of patient age and gender among 40 prescribers in three clinics.***

***Figure S3: Observed prescription proportions by total consultations.*** *Marker colour and shape corresponds to clinic site, dashed horizontal line represent average prescription percentage.*

***Figure S4:* *Caterpillar plot showing prescriber residuals with 95% confidence intervals for log odds of prescribing antibiotics of prescriber only model.*** *The dotted line at zero denotes no difference from the main intercept. A prescriber with a residual above the zero line prescribes antibiotics more frequently than the main intercept and a prescriber with a residual below the zero line prescribes antibiotics less frequently. All prescribers are displayed****.***

***Figure S5:* *Caterpillar plot showing prescriber residuals with 95% confidence intervals for log odds of prescribing antibiotics of adjusted mode.*** *The dotted line at zero denotes no difference from the main intercept. A prescriber with a residual above the zero line prescribes antibiotics more frequently than the main intercept and a prescriber with a residual below the zero line prescribes antibiotics less frequently. All prescribers are displayed****.***
